# Dr. Flint’s Treatise on Diseases of the Heart

**Published:** 1859-10

**Authors:** 


*Dr. Flint's Treatise on Diseases of the Heart.*—This work will be issued by the publishing house of Blanchard & Lea, as soon as this notice appears. It is an octavo of four hundred and sixty-five pages, devoted to the diagnosis, pathology, and treatment of cardiac diseases. There is no work in our language treating of this subject so completely as this; and the importance of these subjects is daily felt by the practitioner. This work will undoubtedly fill a void which has long been felt in this department. An extended notice will appear hereafter.

